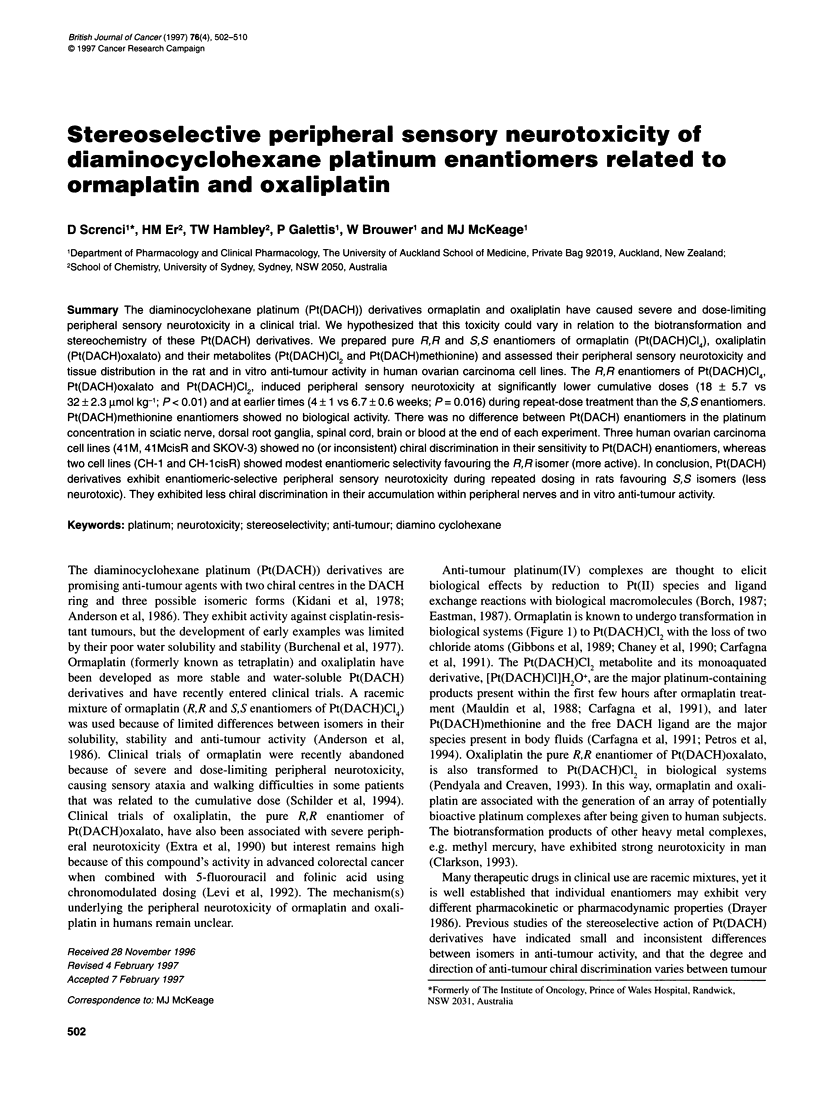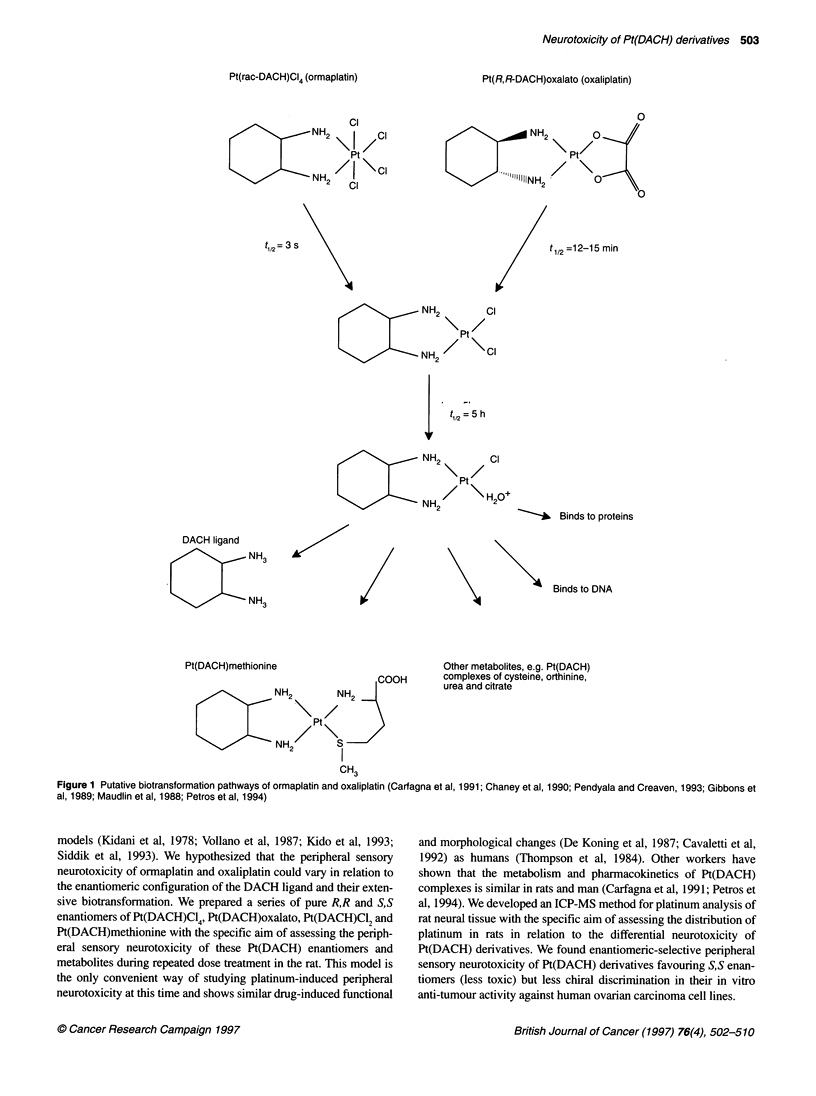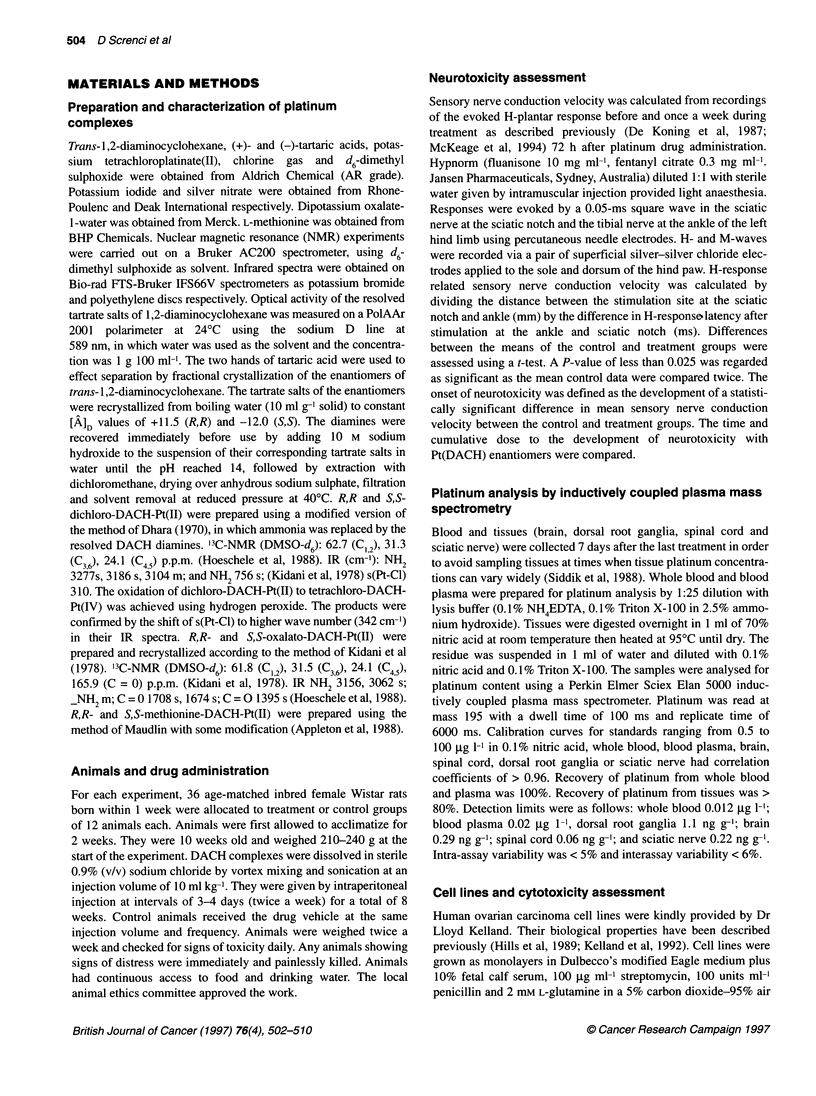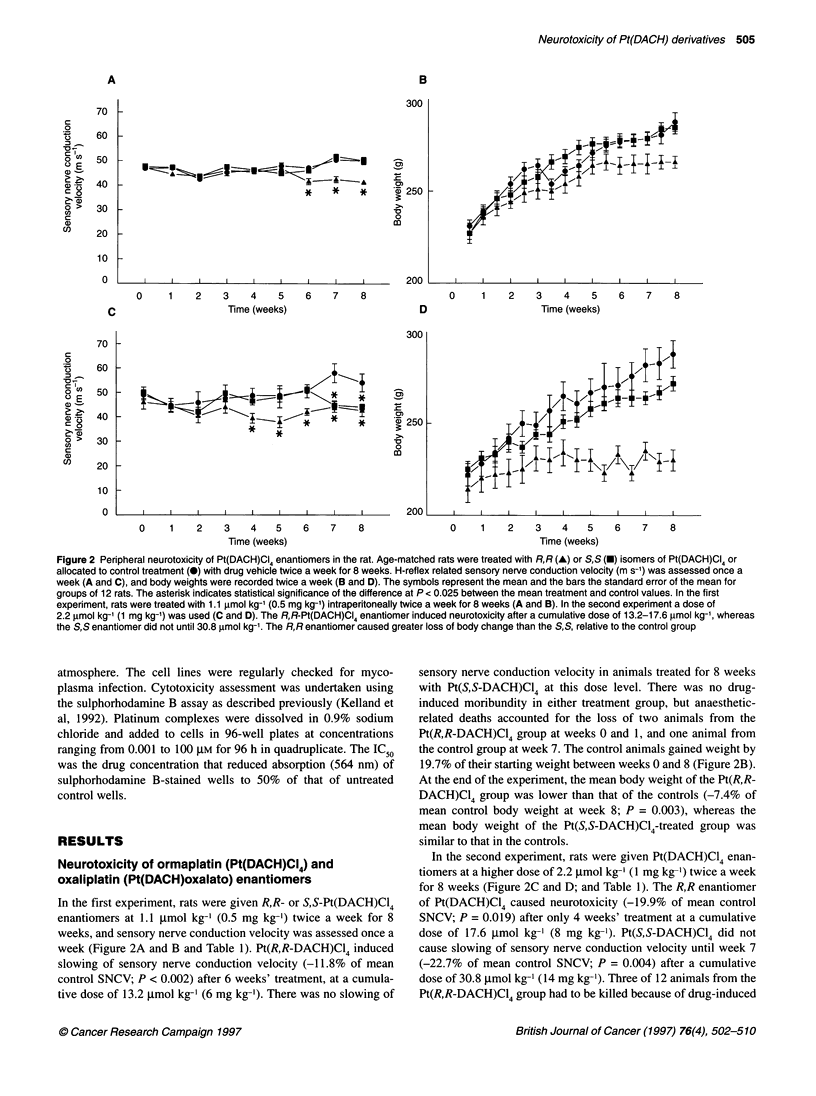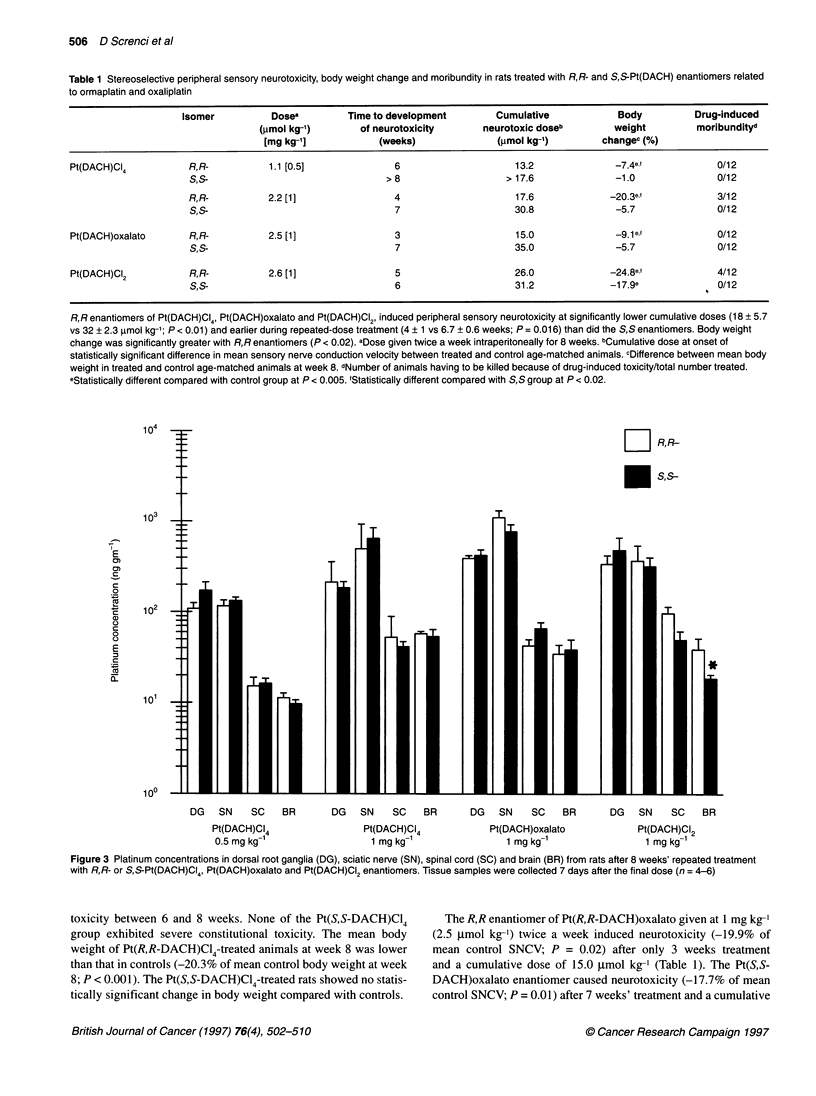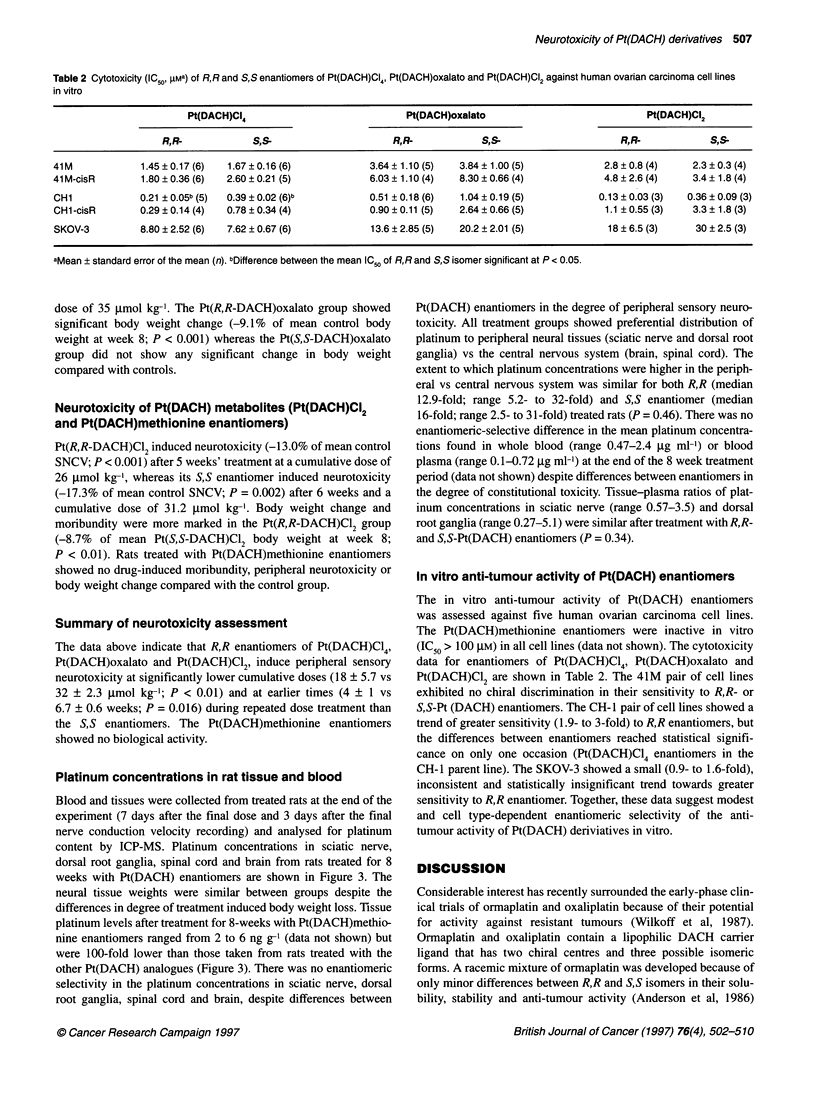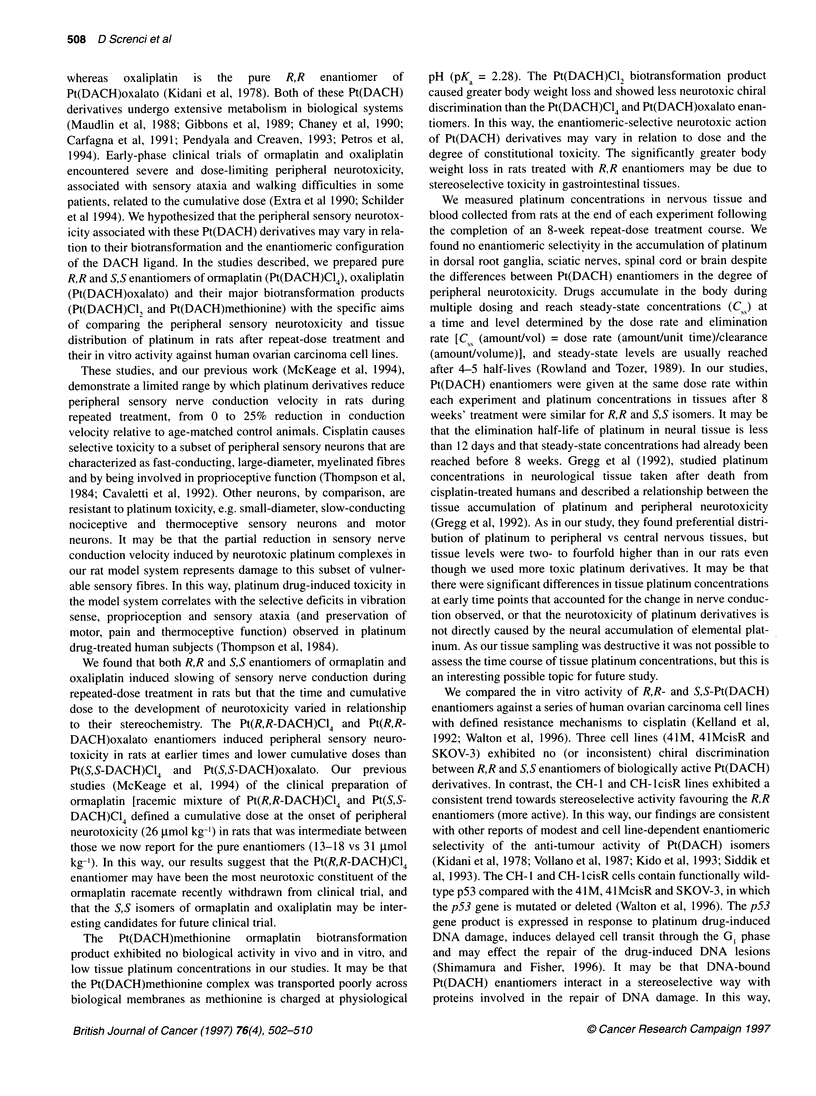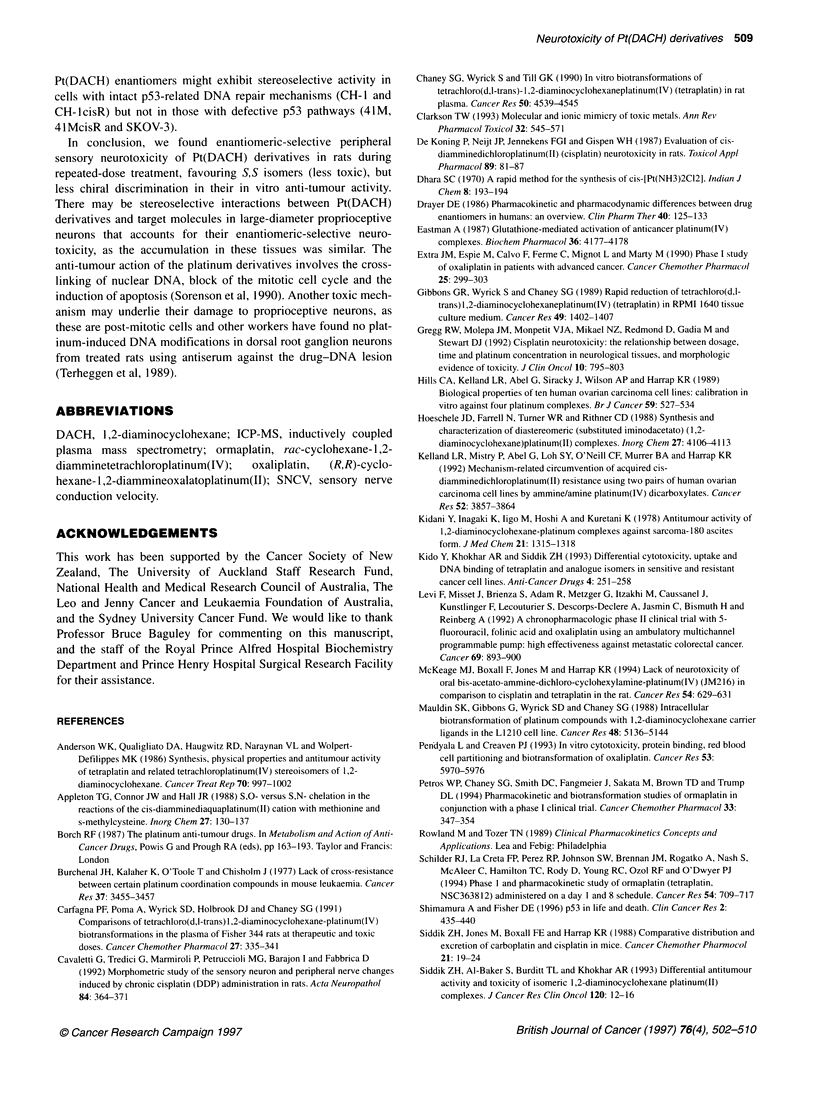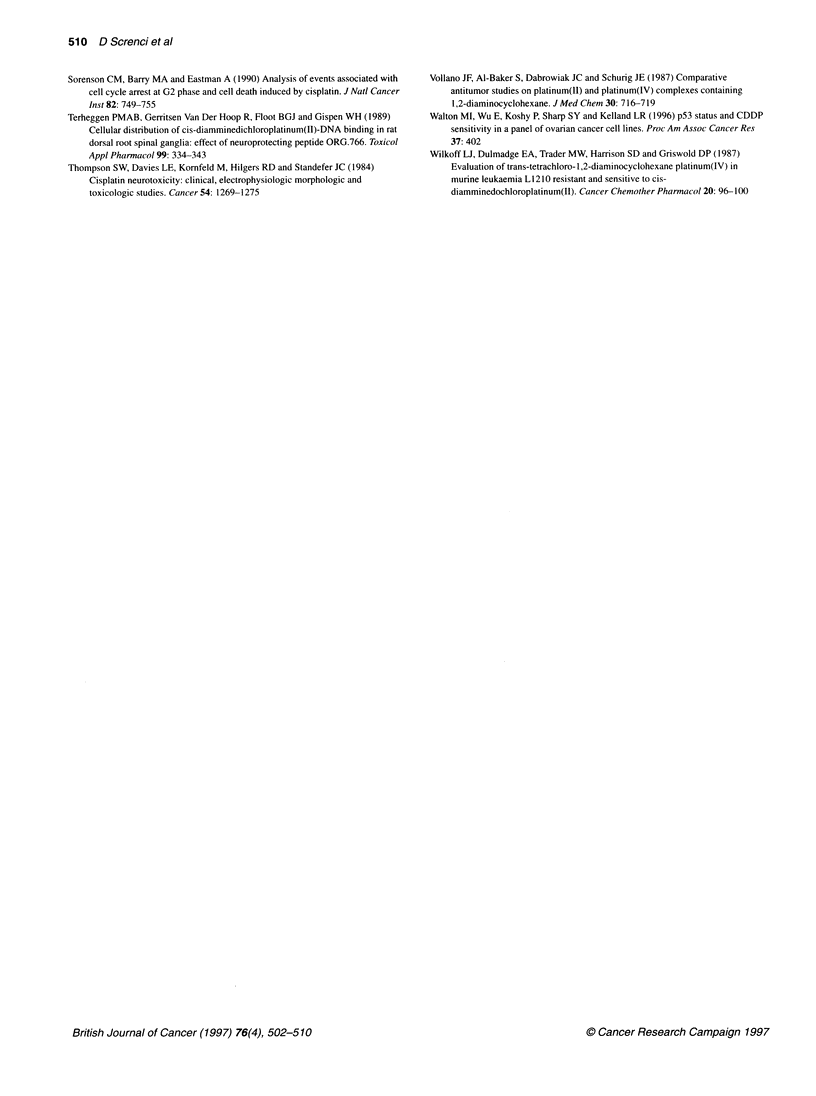# Stereoselective peripheral sensory neurotoxicity of diaminocyclohexane platinum enantiomers related to ormaplatin and oxaliplatin.

**DOI:** 10.1038/bjc.1997.416

**Published:** 1997

**Authors:** D. Screnci, H. M. Er, T. W. Hambley, P. Galettis, W. Brouwer, M. J. McKeage

**Affiliations:** Department of Pharmacology and Clinical Pharmacology, The University of Auckland School of Medicine, New Zealand.

## Abstract

The diaminocyclohexane platinum (Pt(DACH)) derivatives ormaplatin and oxaliplatin have caused severe and dose-limiting peripheral sensory neurotoxicity in a clinical trial. We hypothesized that this toxicity could vary in relation to the biotransformation and stereochemistry of these Pt(DACH) derivatives. We prepared pure R,R and S,S enantiomers of ormaplatin (Pt(DACH)Cl4), oxaliplatin (Pt(DACH)oxalato) and their metabolites (Pt(DACH)Cl2 and Pt(DACH)methionine) and assessed their peripheral sensory neurotoxicity and tissue distribution in the rat and in vitro anti-tumour activity in human ovarian carcinoma cell lines. The R,R enantiomers of Pt(DACH)Cl4, Pt(DACH)oxalato and Pt(DACH)Cl2, induced peripheral sensory neurotoxicity at significantly lower cumulative doses (18 +/- 5.7 vs 32 +/- 2.3 micromol kg(-1); P < 0.01) and at earlier times (4 +/- 1 vs 6.7 +/- 0.6 weeks; P = 0.016) during repeat-dose treatment than the S,S enantiomers. Pt(DACH)methionine enantiomers showed no biological activity. There was no difference between Pt(DACH) enantiomers in the platinum concentration in sciatic nerve, dorsal root ganglia, spinal cord, brain or blood at the end of each experiment. Three human ovarian carcinoma cell lines (41 M, 41 McisR and SKOV-3) showed no (or inconsistent) chiral discrimination in their sensitivity to Pt(DACH) enantiomers, whereas two cell lines (CH-1 and CH-1cisR) showed modest enantiomeric selectivity favouring the R,R isomer (more active). In conclusion, Pt(DACH) derivatives exhibit enantiomeric-selective peripheral sensory neurotoxicity during repeated dosing in rats favouring S,S isomers (less neurotoxic). They exhibited less chiral discrimination in their accumulation within peripheral nerves and in vitro anti-tumour activity.


British Journal of Cancer (1997) 76(4), 502–510
© 1997 Cancer Research Campaign

### Stereoselective peripheral sensory neurotoxicity of diaminocyclohexane platinum enantiomers related to ormaplatin and oxaliplatin

**D Screnci[1]*, HM Er[2], TW Hambley[2], P Galettis[1], W Brouwer[1] and MJ McKeage[1]**

[1]Department of Pharmacology and Clinical Pharmacology, The University of Auckland School of Medicine, Private Bag 92019, Auckland, New Zealand; [2]School of Chemistry, University of Sydney, Sydney, NSW 2050, Australia

**Summary** The diaminocyclohexane platinum (Pt(DACH)) derivatives ormaplatin and oxaliplatin have caused severe and dose-limiting peripheral sensory neurotoxicity in a clinical trial. We hypothesized that this toxicity could vary in relation to the biotransformation and stereochemistry of these Pt(DACH) derivatives. We prepared pure *R,R* and *S,S* enantiomers of ormaplatin (Pt(DACH)$Cl_4$), oxaliplatin (Pt(DACH)oxalato) and their metabolites (Pt(DACH)$Cl_2$ and Pt(DACH)methionine) and assessed their peripheral sensory neurotoxicity and tissue distribution in the rat and in vitro anti-tumour activity in human ovarian carcinoma cell lines. The *R,R* enantiomers of Pt(DACH)$Cl_4$, Pt(DACH)oxalato and Pt(DACH)$Cl_2$, induced peripheral sensory neurotoxicity at significantly lower cumulative doses (18 ± 5.7 vs 32 ± 2.3 μmol $kg^{-1}$; $P < 0.01$) and at earlier times (4 ± 1 vs 6.7 ± 0.6 weeks; $P = 0.016$) during repeat-dose treatment than the *S,S* enantiomers. Pt(DACH)methionine enantiomers showed no biological activity. There was no difference between Pt(DACH) enantiomers in the platinum concentration in sciatic nerve, dorsal root ganglia, spinal cord, brain or blood at the end of each experiment. Three human ovarian carcinoma cell lines (41M, 41McisR and SKOV-3) showed no (or inconsistent) chiral discrimination in their sensitivity to Pt(DACH) enantiomers, whereas two cell lines (CH-1 and CH-1cisR) showed modest enantiomeric selectivity favouring the *R,R* isomer (more active). In conclusion, Pt(DACH) derivatives exhibit enantiomeric-selective peripheral sensory neurotoxicity during repeated dosing in rats favouring *S,S* isomers (less neurotoxic). They exhibited less chiral discrimination in their accumulation within peripheral nerves and in vitro anti-tumour activity.

**Keywords:** platinum; neurotoxicity; stereoselectivity; anti-tumour; diamino cyclohexane

The diaminocyclohexane platinum (Pt(DACH)) derivatives are promising anti-tumour agents with two chiral centres in the DACH ring and three possible isomeric forms (Kidani et al, 1978; Anderson et al, 1986). They exhibit activity against cisplatin-resistant tumours, but the development of early examples was limited by their poor water solubility and stability (Burchenal et al, 1977). Ormaplatin (formerly known as tetraplatin) and oxaliplatin have been developed as more stable and water-soluble Pt(DACH) derivatives and have recently entered clinical trials. A racemic mixture of ormaplatin (*R,R* and *S,S* enantiomers of Pt(DACH)$Cl_4$) was used because of limited differences between isomers in their solubility, stability and anti-tumour activity (Anderson et al, 1986). Clinical trials of ormaplatin were recently abandoned because of severe and dose-limiting peripheral neurotoxicity, causing sensory ataxia and walking difficulties in some patients that was related to the cumulative dose (Schilder et al, 1994). Clinical trials of oxaliplatin, the pure *R,R* enantiomer of Pt(DACH)oxalato, have also been associated with severe peripheral neurotoxicity (Extra et al, 1990) but interest remains high because of this compound's activity in advanced colorectal cancer when combined with 5-fluorouracil and folinic acid using chronomodulated dosing (Levi et al, 1992). The mechanism(s) underlying the peripheral neurotoxicity of ormaplatin and oxaliplatin in humans remain unclear.

*Received 28 November 1996*
*Revised 4 February 1997*
*Accepted 7 February 1997*

*Correspondence to:* MJ McKeage

Anti-tumour platinum(IV) complexes are thought to elicit biological effects by reduction to Pt(II) species and ligand exchange reactions with biological macromolecules (Borch, 1987; Eastman, 1987). Ormaplatin is known to undergo transformation in biological systems (Figure 1) to Pt(DACH)$Cl_2$ with the loss of two chloride atoms (Gibbons et al, 1989; Chaney et al, 1990; Carfagna et al, 1991). The Pt(DACH)$Cl_2$ metabolite and its monoaquated derivative, $[Pt(DACH)Cl]H_2O^+$, are the major platinum-containing products present within the first few hours after ormaplatin treatment (Mauldin et al, 1988; Carfagna et al, 1991), and later Pt(DACH)methionine and the free DACH ligand are the major species present in body fluids (Carfagna et al, 1991; Petros et al, 1994). Oxaliplatin the pure *R,R* enantiomer of Pt(DACH)oxalato, is also transformed to Pt(DACH)$Cl_2$ in biological systems (Pendyala and Creaven, 1993). In this way, ormaplatin and oxaliplatin are associated with the generation of an array of potentially bioactive platinum complexes after being given to human subjects. The biotransformation products of other heavy metal complexes, e.g. methyl mercury, have exhibited strong neurotoxicity in man (Clarkson, 1993).

Many therapeutic drugs in clinical use are racemic mixtures, yet it is well established that individual enantiomers may exhibit very different pharmacokinetic or pharmacodynamic properties (Drayer 1986). Previous studies of the stereoselective action of Pt(DACH) derivatives have indicated small and inconsistent differences between isomers in anti-tumour activity, and that the degree and direction of anti-tumour chiral discrimination varies between tumour

*Formerly of The Institute of Oncology, Prince of Wales Hospital, Randwick, NSW 2031, Australia

**Figure 1** Putative biotransformation pathways of ormaplatin and oxaliplatin (Carfagna et al, 1991; Chaney et al, 1990; Pendyala and Creaven, 1993; Gibbons et al, 1989; Maudlin et al, 1988; Petros et al, 1994)

models (Kidani et al, 1978; Vollano et al, 1987; Kido et al, 1993; Siddik et al, 1993). We hypothesized that the peripheral sensory neurotoxicity of ormaplatin and oxaliplatin could vary in relation to the enantiomeric configuration of the DACH ligand and their extensive biotransformation. We prepared a series of pure *R,R* and *S,S* enantiomers of Pt(DACH)$Cl_4$, Pt(DACH)oxalato, Pt(DACH)$Cl_2$ and Pt(DACH)methionine with the specific aim of assessing the peripheral sensory neurotoxicity of these Pt(DACH) enantiomers and metabolites during repeated dose treatment in the rat. This model is the only convenient way of studying platinum-induced peripheral neurotoxicity at this time and shows similar drug-induced functional and morphological changes (De Koning et al, 1987; Cavaletti et al, 1992) as humans (Thompson et al, 1984). Other workers have shown that the metabolism and pharmacokinetics of Pt(DACH) complexes is similar in rats and man (Carfagna et al, 1991; Petros et al, 1994). We developed an ICP-MS method for platinum analysis of rat neural tissue with the specific aim of assessing the distribution of platinum in rats in relation to the differential neurotoxicity of Pt(DACH) derivatives. We found enantiomeric-selective peripheral sensory neurotoxicity of Pt(DACH) derivatives favouring *S,S* enantiomers (less toxic) but less chiral discrimination in their in vitro anti-tumour activity against human ovarian carcinoma cell lines.

© Cancer Research Campaign 1997

### MATERIALS AND METHODS

### Preparation and characterization of platinum complexes

*Trans*-1,2-diaminocyclohexane, (+)- and (–)-tartaric acids, potassium tetrachloroplatinate(II), chlorine gas and $d_6$-dimethyl sulphoxide were obtained from Aldrich Chemical (AR grade). Potassium iodide and silver nitrate were obtained from Rhone-Poulenc and Deak International respectively. Dipotassium oxalate-1-water was obtained from Merck. L-methionine was obtained from BHP Chemicals. Nuclear magnetic resonance (NMR) experiments were carried out on a Bruker AC200 spectrometer, using $d_6$-dimethyl sulphoxide as solvent. Infrared spectra were obtained on Bio-rad FTS-Bruker IFS66V spectrometers as potassium bromide and polyethylene discs respectively. Optical activity of the resolved tartrate salts of 1,2-diaminocyclohexane was measured on a PolAAr 2001 polarimeter at 24°C using the sodium D line at 589 nm, in which water was used as the solvent and the concentration was 1 g 100 ml$^{-1}$. The two hands of tartaric acid were used to effect separation by fractional crystallization of the enantiomers of *trans*-1,2-diaminocyclohexane. The tartrate salts of the enantiomers were recrystallized from boiling water (10 ml g$^{-1}$ solid) to constant $[\hat{A}]_D$ values of +11.5 (*R,R*) and –12.0 (*S,S*). The diamines were recovered immediately before use by adding 10 M sodium hydroxide to the suspension of their corresponding tartrate salts in water until the pH reached 14, followed by extraction with dichloromethane, drying over anhydrous sodium sulphate, filtration and solvent removal at reduced pressure at 40°C. *R,R* and *S,S*-dichloro-DACH-Pt(II) were prepared using a modified version of the method of Dhara (1970), in which ammonia was replaced by the resolved DACH diamines. $^{13}$C-NMR (DMSO-$d_6$): 62.7 ($C_{1,2}$), 31.3 ($C_{3,6}$), 24.1 ($C_{4,5}$) p.p.m. (Hoeschele et al, 1988). IR (cm$^{-1}$): $NH_2$ 3277s, 3186 s, 3104 m; and $NH_2$ 756 s; (Kidani et al, 1978) s(Pt-Cl) 310. The oxidation of dichloro-DACH-Pt(II) to tetrachloro-DACH-Pt(IV) was achieved using hydrogen peroxide. The products were confirmed by the shift of s(Pt-Cl) to higher wave number (342 cm$^{-1}$) in their IR spectra. *R,R*- and *S,S*-oxalato-DACH-Pt(II) were prepared and recrystallized according to the method of Kidani et al (1978). $^{13}$C-NMR (DMSO-$d_6$): 61.8 ($C_{1,2}$), 31.5 ($C_{3,6}$), 24.1 ($C_{4,5}$), 165.9 (C = 0) p.p.m. (Kidani et al, 1978). IR $NH_2$ 3156, 3062 s; _$NH_2$ m; C = 0 1708 s, 1674 s; C = O 1395 s (Hoeschele et al, 1988). *R,R*- and *S,S*-methionine-DACH-Pt(II) were prepared using the method of Maudlin with some modification (Appleton et al, 1988).

### Animals and drug administration

For each experiment, 36 age-matched inbred female Wistar rats born within 1 week were allocated to treatment or control groups of 12 animals each. Animals were first allowed to acclimatize for 2 weeks. They were 10 weeks old and weighed 210–240 g at the start of the experiment. DACH complexes were dissolved in sterile 0.9% (v/v) sodium chloride by vortex mixing and sonication at an injection volume of 10 ml kg$^{-1}$. They were given by intraperitoneal injection at intervals of 3–4 days (twice a week) for a total of 8 weeks. Control animals received the drug vehicle at the same injection volume and frequency. Animals were weighed twice a week and checked for signs of toxicity daily. Any animals showing signs of distress were immediately and painlessly killed. Animals had continuous access to food and drinking water. The local animal ethics committee approved the work.

### Neurotoxicity assessment

Sensory nerve conduction velocity was calculated from recordings of the evoked H-plantar response before and once a week during treatment as described previously (De Koning et al, 1987; McKeage et al, 1994) 72 h after platinum drug administration. Hypnorm (fluanisone 10 mg ml$^{-1}$, fentanyl citrate 0.3 mg ml$^{-1}$. Jansen Pharmaceuticals, Sydney, Australia) diluted 1:1 with sterile water given by intramuscular injection provided light anaesthesia. Responses were evoked by a 0.05-ms square wave in the sciatic nerve at the sciatic notch and the tibial nerve at the ankle of the left hind limb using percutaneous needle electrodes. H- and M-waves were recorded via a pair of superficial silver–silver chloride electrodes applied to the sole and dorsum of the hind paw. H-response related sensory nerve conduction velocity was calculated by dividing the distance between the stimulation site at the sciatic notch and ankle (mm) by the difference in H-response latency after stimulation at the ankle and sciatic notch (ms). Differences between the means of the control and treatment groups were assessed using a *t*-test. A *P*-value of less than 0.025 was regarded as significant as the mean control data were compared twice. The onset of neurotoxicity was defined as the development of a statistically significant difference in mean sensory nerve conduction velocity between the control and treatment groups. The time and cumulative dose to the development of neurotoxicity with Pt(DACH) enantiomers were compared.

### Platinum analysis by inductively coupled plasma mass spectrometry

Blood and tissues (brain, dorsal root ganglia, spinal cord and sciatic nerve) were collected 7 days after the last treatment in order to avoid sampling tissues at times when tissue platinum concentrations can vary widely (Siddik et al, 1988). Whole blood and blood plasma were prepared for platinum analysis by 1:25 dilution with lysis buffer (0.1% $NH_4$EDTA, 0.1% Triton X-100 in 2.5% ammonium hydroxide). Tissues were digested overnight in 1 ml of 70% nitric acid at room temperature then heated at 95°C until dry. The residue was suspended in 1 ml of water and diluted with 0.1% nitric acid and 0.1% Triton X-100. The samples were analysed for platinum content using a Perkin Elmer Sciex Elan 5000 inductively coupled plasma mass spectrometer. Platinum was read at mass 195 with a dwell time of 100 ms and replicate time of 6000 ms. Calibration curves for standards ranging from 0.5 to 100 μg l$^{-1}$ in 0.1% nitric acid, whole blood, blood plasma, brain, spinal cord, dorsal root ganglia or sciatic nerve had correlation coefficients of > 0.96. Recovery of platinum from whole blood and plasma was 100%. Recovery of platinum from tissues was > 80%. Detection limits were as follows: whole blood 0.012 μg l$^{-1}$; blood plasma 0.02 μg 1$^{-1}$, dorsal root ganglia 1.1 ng g$^{-1}$; brain 0.29 ng g$^{-1}$; spinal cord 0.06 ng g$^{-1}$; and sciatic nerve 0.22 ng g$^{-1}$. Intra-assay variability was < 5% and interassay variability < 6%.

### Cell lines and cytotoxicity assessment

Human ovarian carcinoma cell lines were kindly provided by Dr Lloyd Kelland. Their biological properties have been described previously (Hills et al, 1989; Kelland et al, 1992). Cell lines were grown as monolayers in Dulbecco's modified Eagle medium plus 10% fetal calf serum, 100 μg ml$^{-1}$ streptomycin, 100 units ml$^{-1}$ penicillin and 2 mM L-glutamine in a 5% carbon dioxide–95% air

 © Cancer Research Campaign 1997

**Figure 2** Peripheral neurotoxicity of $Pt(DACH)Cl_4$ enantiomers in the rat. Age-matched rats were treated with *R,R* (▲) or *S,S* (■) isomers of $Pt(DACH)Cl_4$ or allocated to control treatment (●) with drug vehicle twice a week for 8 weeks. H-reflex related sensory nerve conduction velocity (m $s^{-1}$) was assessed once a week (**A** and **C**), and body weights were recorded twice a week (**B** and **D**). The symbols represent the mean and the bars the standard error of the mean for groups of 12 rats. The asterisk indicates statistical significance of the difference at $P < 0.025$ between the mean treatment and control values. In the first experiment, rats were treated with 1.1 μmol $kg^{-1}$ (0.5 mg $kg^{-1}$) intraperitoneally twice a week for 8 weeks (**A** and **B**). In the second experiment a dose of 2.2 μmol $kg^{-1}$ (1 mg $kg^{-1}$) was used (**C** and **D**). The *R,R*-$Pt(DACH)Cl_4$ enantiomer induced neurotoxicity after a cumulative dose of 13.2–17.6 μmol $kg^{-1}$, whereas the *S,S* enantiomer did not until 30.8 μmol $kg^{-1}$. The *R,R* enantiomer caused greater loss of body change than the *S,S*, relative to the control group atmosphere. The cell lines were regularly checked for mycoplasma infection. Cytotoxicity assessment was undertaken using the sulphorhodamine B assay as described previously (Kelland et al, 1992). Platinum complexes were dissolved in 0.9% sodium chloride and added to cells in 96-well plates at concentrations ranging from 0.001 to 100 μM for 96 h in quadruplicate. The $IC_{50}$ was the drug concentration that reduced absorption (564 nm) of sulphorhodamine B-stained wells to 50% of that of untreated control wells.

### RESULTS

### Neurotoxicity of ormaplatin ($Pt(DACH)Cl_4$) and oxaliplatin (Pt(DACH)oxalato) enantiomers

In the first experiment, rats were given *R,R*- or *S,S*-$Pt(DACH)Cl_4$ enantiomers at 1.1 μmol $kg^{-1}$ (0.5 mg $kg^{-1}$) twice a week for 8 weeks, and sensory nerve conduction velocity was assessed once a week (Figure 2A and B and Table 1). Pt(*R,R*-DACH)$Cl_4$ induced slowing of sensory nerve conduction velocity (–11.8% of mean control SNCV; $P < 0.002$) after 6 weeks' treatment, at a cumulative dose of 13.2 μmol $kg^{-1}$ (6 mg $kg^{-1}$). There was no slowing of sensory nerve conduction velocity in animals treated for 8 weeks with Pt(*S,S*-DACH)$Cl_4$ at this dose level. There was no drug-induced moribundity in either treatment group, but anaesthetic-related deaths accounted for the loss of two animals from the Pt(*R,R*-DACH)$Cl_4$ group at weeks 0 and 1, and one animal from the control group at week 7. The control animals gained weight by 19.7% of their starting weight between weeks 0 and 8 (Figure 2B). At the end of the experiment, the mean body weight of the Pt(*R,R*-DACH)$Cl_4$ group was lower than that of the controls (–7.4% of mean control body weight at week 8; $P = 0.003$), whereas the mean body weight of the Pt(*S,S*-DACH)$Cl_4$-treated group was similar to that in the controls.

In the second experiment, rats were given $Pt(DACH)Cl_4$ enantiomers at a higher dose of 2.2 μmol $kg^{-1}$ (1 mg $kg^{-1}$) twice a week for 8 weeks (Figure 2C and D; and Table 1). The *R,R* enantiomer of $Pt(DACH)Cl_4$ caused neurotoxicity (–19.9% of mean control SNCV; $P = 0.019$) after only 4 weeks' treatment at a cumulative dose of 17.6 μmol $kg^{-1}$ (8 mg $kg^{-1}$). Pt(*S,S*-DACH)$Cl_4$ did not cause slowing of sensory nerve conduction velocity until week 7 (–22.7% of mean control SNCV; $P = 0.004$) after a cumulative dose of 30.8 μmol $kg^{-1}$ (14 mg $kg^{-1}$). Three of 12 animals from the Pt(*R,R*-DACH)$Cl_4$ group had to be killed because of drug-induced

**Table 1** Stereoselective peripheral sensory neurotoxicity, body weight change and moribundity in rats treated with *R,R*- and *S,S*-Pt(DACH) enantiomers related to ormaplatin and oxaliplatin

| | Isomer | Dose[a] (μmol kg⁻¹) [mg kg⁻¹] | Time to development of neurotoxicity (weeks) | Cumulative neurotoxic dose[b] (μmol kg⁻¹) | Body weight change[c] (%) | Drug-induced moribundity[d] |
|---|---|---|---|---|---|---|
| $Pt(DACH)Cl_4$ | *R,R*- | 1.1 [0.5] | 6 | 13.2 | –7.4[e,f] | 0/12 |
| | *S,S*- | | > 8 | > 17.6 | –1.0 | 0/12 |
| | *R,R*- | 2.2 [1] | 4 | 17.6 | –20.3[e,f] | 3/12 |
| | *S,S*- | | 7 | 30.8 | –5.7 | 0/12 |
| Pt(DACH)oxalato | *R,R*- | 2.5 [1] | 3 | 15.0 | –9.1[e,f] | 0/12 |
| | *S,S*- | | 7 | 35.0 | –5.7 | 0/12 |
| $Pt(DACH)Cl_2$ | *R,R*- | 2.6 [1] | 5 | 26.0 | –24.8[e,f] | 4/12 |
| | *S,S*- | | 6 | 31.2 | –17.9[e] | 0/12 |

*R,R* enantiomers of $Pt(DACH)Cl_4$, Pt(DACH)oxalato and $Pt(DACH)Cl_2$, induced peripheral sensory neurotoxicity at significantly lower cumulative doses (18 ± 5.7 vs 32 ± 2.3 μmol kg⁻¹; $P < 0.01$) and earlier during repeated-dose treatment (4 ± 1 vs 6.7 ± 0.6 weeks; $P = 0.016$) than did the *S,S* enantiomers. Body weight change was significantly greater with *R,R* enantiomers ($P < 0.02$). [a]Dose given twice a week intraperitoneally for 8 weeks. [b]Cumulative dose at onset of statistically significant difference in mean sensory nerve conduction velocity between treated and control age-matched animals. [c]Difference between mean body weight in treated and control age-matched animals at week 8. [d]Number of animals having to be killed because of drug-induced toxicity/total number treated. [e]Statistically different compared with control group at $P < 0.005$. [f]Statistically different compared with *S,S* group at $P < 0.02$.

**Figure 3** Platinum concentrations in dorsal root ganglia (DG), sciatic nerve (SN), spinal cord (SC) and brain (BR) from rats after 8 weeks' repeated treatment with *R,R*- or *S,S*-$Pt(DACH)Cl_4$, Pt(DACH)oxalato and $Pt(DACH)Cl_2$ enantiomers. Tissue samples were collected 7 days after the final dose ($n$ = 4–6)

toxicity between 6 and 8 weeks. None of the Pt(*S,S*-DACH)$Cl_4$ group exhibited severe constitutional toxicity. The mean body weight of Pt(*R,R*-DACH)$Cl_4$-treated animals at week 8 was lower than that in controls (–20.3% of mean control body weight at week 8; $P < 0.001$). The Pt(*S,S*-DACH)$Cl_4$-treated rats showed no statistically significant change in body weight compared with controls.

The *R,R* enantiomer of Pt(*R,R*-DACH)oxalato given at 1 mg kg⁻¹ (2.5 μmol kg⁻¹) twice a week induced neurotoxicity (–19.9% of mean control SNCV; $P = 0.02$) after only 3 weeks treatment and a cumulative dose of 15.0 μmol kg⁻¹ (Table 1). The Pt(*S,S*-DACH)oxalato enantiomer caused neurotoxicity (–17.7% of mean control SNCV; $P = 0.01$) after 7 weeks' treatment and a cumulative

© Cancer Research Campaign 1997

**Table 2** Cytotoxicity ($IC_{50}$, μM[a]) of *R,R* and *S,S* enantiomers of $Pt(DACH)Cl_4$, Pt(DACH)oxalato and $Pt(DACH)Cl_2$ against human ovarian carcinoma cell lines in vitro

| | $Pt(DACH)Cl_4$ | | Pt(DACH)oxalato | | $Pt(DACH)Cl_2$ | |
|---|---|---|---|---|---|---|
| | *R,R-* | *S,S-* | *R,R-* | *S,S-* | *R,R-* | *S,S-* |
| 41M | 1.45 ± 0.17 (6) | 1.67 ± 0.16 (6) | 3.64 ± 1.10 (5) | 3.84 ± 1.00 (5) | 2.8 ± 0.8 (4) | 2.3 ± 0.3 (4) |
| 41M-cisR | 1.80 ± 0.36 (6) | 2.60 ± 0.21 (5) | 6.03 ± 1.10 (4) | 8.30 ± 0.66 (4) | 4.8 ± 2.6 (4) | 3.4 ± 1.8 (4) |
| CH1 | 0.21 ± 0.05[b] (5) | 0.39 ± 0.02 (6)[b] | 0.51 ± 0.18 (6) | 1.04 ± 0.19 (5) | 0.13 ± 0.03 (3) | 0.36 ± 0.09 (3) |
| CH1-cisR | 0.29 ± 0.14 (4) | 0.78 ± 0.34 (4) | 0.90 ± 0.11 (5) | 2.64 ± 0.66 (5) | 1.1 ± 0.55 (3) | 3.3 ± 1.8 (3) |
| SKOV-3 | 8.80 ± 2.52 (6) | 7.62 ± 0.67 (6) | 13.6 ± 2.85 (5) | 20.2 ± 2.01 (5) | 18 ± 6.5 (3) | 30 ± 2.5 (3) |

[a]Mean ± standard error of the mean (*n*). [b]Difference between the mean $IC_{50}$ of *R,R* and *S,S* isomer significant at $P < 0.05$.

dose of 35 μmol $kg^{-1}$. The Pt(*R,R*-DACH)oxalato group showed significant body weight change (–9.1% of mean control body weight at week 8; $P < 0.001$) whereas the Pt(*S,S*-DACH)oxalato group did not show any significant change in body weight compared with controls.

### Neurotoxicity of Pt(DACH) metabolites ($Pt(DACH)Cl_2$ and Pt(DACH)methionine enantiomers)

Pt(*R,R*-DACH)$Cl_2$ induced neurotoxicity (–13.0% of mean control SNCV; $P < 0.001$) after 5 weeks' treatment at a cumulative dose of 26 μmol $kg^{-1}$, whereas its *S,S* enantiomer induced neurotoxicity (–17.3% of mean control SNCV; $P = 0.002$) after 6 weeks and a cumulative dose of 31.2 μmol $kg^{-1}$. Body weight change and moribundity were more marked in the Pt(*R,R*-DACH)$Cl_2$ group (–8.7% of mean Pt(*S,S*-DACH)$Cl_2$ body weight at week 8; $P < 0.01$). Rats treated with Pt(DACH)methionine enantiomers showed no drug-induced moribundity, peripheral neurotoxicity or body weight change compared with the control group.

### Summary of neurotoxicity assessment

The data above indicate that *R,R* enantiomers of $Pt(DACH)Cl_4$, Pt(DACH)oxalato and $Pt(DACH)Cl_2$, induce peripheral sensory neurotoxicity at significantly lower cumulative doses (18 ± 5.7 vs 32 ± 2.3 μmol $kg^{-1}$; $P < 0.01$) and at earlier times (4 ± 1 vs 6.7 ± 0.6 weeks; $P = 0.016$) during repeated dose treatment than the *S,S* enantiomers. The Pt(DACH)methionine enantiomers showed no biological activity.

### Platinum concentrations in rat tissue and blood

Blood and tissues were collected from treated rats at the end of the experiment (7 days after the final dose and 3 days after the final nerve conduction velocity recording) and analysed for platinum content by ICP-MS. Platinum concentrations in sciatic nerve, dorsal root ganglia, spinal cord and brain from rats treated for 8 weeks with Pt(DACH) enantiomers are shown in Figure 3. The neural tissue weights were similar between groups despite the differences in degree of treatment induced body weight loss. Tissue platinum levels after treatment for 8-weeks with Pt(DACH)methionine enantiomers ranged from 2 to 6 ng $g^{-1}$ (data not shown) but were 100-fold lower than those taken from rats treated with the other Pt(DACH) analogues (Figure 3). There was no enantiomeric selectivity in the platinum concentrations in sciatic nerve, dorsal root ganglia, spinal cord and brain, despite differences between Pt(DACH) enantiomers in the degree of peripheral sensory neurotoxicity. All treatment groups showed preferential distribution of platinum to peripheral neural tissues (sciatic nerve and dorsal root ganglia) vs the central nervous system (brain, spinal cord). The extent to which platinum concentrations were higher in the peripheral vs central nervous system was similar for both *R,R* (median 12.9-fold; range 5.2- to 32-fold) and *S,S* enantiomer (median 16-fold; range 2.5- to 31-fold) treated rats ($P = 0.46$). There was no enantiomeric-selective difference in the mean platinum concentrations found in whole blood (range 0.47–2.4 μg $ml^{-1}$) or blood plasma (range 0.1–0.72 μg $ml^{-1}$) at the end of the 8 week treatment period (data not shown) despite differences between enantiomers in the degree of constitutional toxicity. Tissue–plasma ratios of platinum concentrations in sciatic nerve (range 0.57–3.5) and dorsal root ganglia (range 0.27–5.1) were similar after treatment with *R,R*- and *S,S*-Pt(DACH) enantiomers ($P = 0.34$).

### In vitro anti-tumour activity of Pt(DACH) enantiomers

The in vitro anti-tumour activity of Pt(DACH) enantiomers was assessed against five human ovarian carcinoma cell lines. The Pt(DACH)methionine enantiomers were inactive in vitro ($IC_{50} > 100$ μM) in all cell lines (data not shown). The cytotoxicity data for enantiomers of $Pt(DACH)Cl_4$, Pt(DACH)oxalato and $Pt(DACH)Cl_2$ are shown in Table 2. The 41M pair of cell lines exhibited no chiral discrimination in their sensitivity to *R,R*- or *S,S*-Pt (DACH) enantiomers. The CH-1 pair of cell lines showed a trend of greater sensitivity (1.9- to 3-fold) to *R,R* enantiomers, but the differences between enantiomers reached statistical significance on only one occasion ($Pt(DACH)Cl_4$ enantiomers in the CH-1 parent line). The SKOV-3 showed a small (0.9- to 1.6-fold), inconsistent and statistically insignificant trend towards greater sensitivity to *R,R* enantiomer. Together, these data suggest modest and cell type-dependent enantiomeric selectivity of the anti-tumour activity of Pt(DACH) deriviatives in vitro.

### DISCUSSION

Considerable interest has recently surrounded the early-phase clinical trials of ormaplatin and oxaliplatin because of their potential for activity against resistant tumours (Wilkoff et al, 1987). Ormaplatin and oxaliplatin contain a lipophilic DACH carrier ligand that has two chiral centres and three possible isomeric forms. A racemic mixture of ormaplatin was developed because of only minor differences between *R,R* and *S,S* isomers in their solubility, stability and anti-tumour activity (Anderson et al, 1986)

© Cancer Research Campaign 1997 

whereas oxaliplatin is the pure *R,R* enantiomer of Pt(DACH)oxalato (Kidani et al, 1978). Both of these Pt(DACH) derivatives undergo extensive metabolism in biological systems (Maudlin et al, 1988; Gibbons et al, 1989; Chaney et al, 1990; Carfagna et al, 1991; Pendyala and Creaven, 1993; Petros et al, 1994). Early-phase clinical trials of ormaplatin and oxaliplatin encountered severe and dose-limiting peripheral neurotoxicity, associated with sensory ataxia and walking difficulties in some patients, related to the cumulative dose (Extra et al 1990; Schilder et al 1994). We hypothesized that the peripheral sensory neurotoxicity associated with these Pt(DACH) derivatives may vary in relation to their biotransformation and the enantiomeric configuration of the DACH ligand. In the studies described, we prepared pure *R,R* and *S,S* enantiomers of ormaplatin ($Pt(DACH)Cl_4$), oxaliplatin (Pt(DACH)oxalato) and their major biotransformation products ($Pt(DACH)Cl_2$ and Pt(DACH)methionine) with the specific aims of comparing the peripheral sensory neurotoxicity and tissue distribution of platinum in rats after repeat-dose treatment and their in vitro activity against human ovarian carcinoma cell lines.

These studies, and our previous work (McKeage et al, 1994), demonstrate a limited range by which platinum derivatives reduce peripheral sensory nerve conduction velocity in rats during repeated treatment, from 0 to 25% reduction in conduction velocity relative to age-matched control animals. Cisplatin causes selective toxicity to a subset of peripheral sensory neurons that are characterized as fast-conducting, large-diameter, myelinated fibres and by being involved in proprioceptive function (Thompson et al, 1984; Cavaletti et al, 1992). Other neurons, by comparison, are resistant to platinum toxicity, e.g. small-diameter, slow-conducting nociceptive and thermoceptive sensory neurons and motor neurons. It may be that the partial reduction in sensory nerve conduction velocity induced by neurotoxic platinum complexes in our rat model system represents damage to this subset of vulnerable sensory fibres. In this way, platinum drug-induced toxicity in the model system correlates with the selective deficits in vibration sense, proprioception and sensory ataxia (and preservation of motor, pain and thermoceptive function) observed in platinum drug-treated human subjects (Thompson et al, 1984).

We found that both *R,R* and *S,S* enantiomers of ormaplatin and oxaliplatin induced slowing of sensory nerve conduction during repeated-dose treatment in rats but that the time and cumulative dose to the development of neurotoxicity varied in relationship to their stereochemistry. The Pt(*R,R*-DACH)$Cl_4$ and Pt(*R,R*-DACH)oxalato enantiomers induced peripheral sensory neurotoxicity in rats at earlier times and lower cumulative doses than Pt(*S,S*-DACH)$Cl_4$ and Pt(*S,S*-DACH)oxalato. Our previous studies (McKeage et al, 1994) of the clinical preparation of ormaplatin [racemic mixture of Pt(*R,R*-DACH)$Cl_4$ and Pt(*S,S*-DACH)$Cl_4$ defined a cumulative dose at the onset of peripheral neurotoxicity (26 $\mu$mol $kg^{-1}$) in rats that was intermediate between those we now report for the pure enantiomers (13–18 vs 31 $\mu$mol $kg^{-1}$). In this way, our results suggest that the Pt(*R,R*-DACH)$Cl_4$ enantiomer may have been the most neurotoxic constituent of the ormaplatin racemate recently withdrawn from clinical trial, and that the *S,S* isomers of ormaplatin and oxaliplatin may be interesting candidates for future clinical trial.

The Pt(DACH)methionine ormaplatin biotransformation product exhibited no biological activity in vivo and in vitro, and low tissue platinum concentrations in our studies. It may be that the Pt(DACH)methionine complex was transported poorly across biological membranes as methionine is charged at physiological pH ($pK_a$ = 2.28). The $Pt(DACH)Cl_2$ biotransformation product caused greater body weight loss and showed less neurotoxic chiral discrimination than the $Pt(DACH)Cl_4$ and Pt(DACH)oxalato enantiomers. In this way, the enantiomeric-selective neurotoxic action of Pt(DACH) derivatives may vary in relation to dose and the degree of constitutional toxicity. The significantly greater body weight loss in rats treated with *R,R* enantiomers may be due to stereoselective toxicity in gastrointestinal tissues.

We measured platinum concentrations in nervous tissue and blood collected from rats at the end of each experiment following the completion of an 8-week repeat-dose treatment course. We found no enantiomeric selectivity in the accumulation of platinum in dorsal root ganglia, sciatic nerves, spinal cord or brain despite the differences between Pt(DACH) enantiomers in the degree of peripheral neurotoxicity. Drugs accumulate in the body during multiple dosing and reach steady-state concentrations ($C_{ss}$) at a time and level determined by the dose rate and elimination rate [$C_{ss}$ (amount/vol) = dose rate (amount/unit time)/clearance (amount/volume)], and steady-state levels are usually reached after 4–5 half-lives (Rowland and Tozer, 1989). In our studies, Pt(DACH) enantiomers were given at the same dose rate within each experiment and platinum concentrations in tissues after 8 weeks' treatment were similar for *R,R* and *S,S* isomers. It may be that the elimination half-life of platinum in neural tissue is less than 12 days and that steady-state concentrations had already been reached before 8 weeks. Gregg et al (1992), studied platinum concentrations in neurological tissue taken after death from cisplatin-treated humans and described a relationship between the tissue accumulation of platinum and peripheral neurotoxicity (Gregg et al, 1992). As in our study, they found preferential distribution of platinum to peripheral vs central nervous tissues, but tissue levels were two- to fourfold higher than in our rats even though we used more toxic platinum derivatives. It may be that there were significant differences in tissue platinum concentrations at early time points that accounted for the change in nerve conduction observed, or that the neurotoxicity of platinum derivatives is not directly caused by the neural accumulation of elemental platinum. As our tissue sampling was destructive it was not possible to assess the time course of tissue platinum concentrations, but this is an interesting possible topic for future study.

We compared the in vitro activity of *R,R*- and *S,S*-Pt(DACH) enantiomers against a series of human ovarian carcinoma cell lines with defined resistance mechanisms to cisplatin (Kelland et al, 1992; Walton et al, 1996). Three cell lines (41M, 41McisR and SKOV-3) exhibited no (or inconsistent) chiral discrimination between *R,R* and *S,S* enantiomers of biologically active Pt(DACH) derivatives. In contrast, the CH-1 and CH-1cisR lines exhibited a consistent trend towards stereoselective activity favouring the *R,R* enantiomers (more active). In this way, our findings are consistent with other reports of modest and cell line-dependent enantiomeric selectivity of the anti-tumour activity of Pt(DACH) isomers (Kidani et al, 1978; Vollano et al, 1987; Kido et al, 1993; Siddik et al, 1993). The CH-1 and CH-1cisR cells contain functionally wild-type p53 compared with the 41M, 41McisR and SKOV-3, in which the *p53* gene is mutated or deleted (Walton et al, 1996). The *p53* gene product is expressed in response to platinum drug-induced DNA damage, induces delayed cell transit through the $G_1$ phase and may effect the repair of the drug-induced DNA lesions (Shimamura and Fisher, 1996). It may be that DNA-bound Pt(DACH) enantiomers interact in a stereoselective way with proteins involved in the repair of DNA damage. In this way,

 © Cancer Research Campaign 1997

Pt(DACH) enantiomers might exhibit stereoselective activity in cells with intact p53-related DNA repair mechanisms (CH-1 and CH-1cisR) but not in those with defective p53 pathways (41M, 41McisR and SKOV-3).

In conclusion, we found enantiomeric-selective peripheral sensory neurotoxicity of Pt(DACH) derivatives in rats during repeated-dose treatment, favouring *S,S* isomers (less toxic), but less chiral discrimination in their in vitro anti-tumour activity. There may be stereoselective interactions between Pt(DACH) derivatives and target molecules in large-diameter proprioceptive neurons that accounts for their enantiomeric-selective neurotoxicity, as the accumulation in these tissues was similar. The anti-tumour action of the platinum derivatives involves the cross-linking of nuclear DNA, block of the mitotic cell cycle and the induction of apoptosis (Sorenson et al, 1990). Another toxic mechanism may underlie their damage to proprioceptive neurons, as these are post-mitotic cells and other workers have found no platinum-induced DNA modifications in dorsal root ganglion neurons from treated rats using antiserum against the drug–DNA lesion (Terheggen et al, 1989).

### ABBREVIATIONS

DACH, 1,2-diaminocyclohexane; ICP-MS, inductively coupled plasma mass spectrometry; ormaplatin, *rac*-cyclohexane-1,2-diamminetetrachloroplatinum(IV); oxaliplatin, (*R,R*)-cyclohexane-1,2-diammineoxalatoplatinum(II); SNCV, sensory nerve conduction velocity.

### ACKNOWLEDGEMENTS

This work has been supported by the Cancer Society of New Zealand, The University of Auckland Staff Research Fund, National Health and Medical Research Council of Australia, The Leo and Jenny Cancer and Leukaemia Foundation of Australia, and the Sydney University Cancer Fund. We would like to thank Professor Bruce Baguley for commenting on this manuscript, and the staff of the Royal Prince Alfred Hospital Biochemistry Department and Prince Henry Hospital Surgical Research Facility for their assistance.

### REFERENCES

Anderson WK, Qualigliato DA, Haugwitz RD, Naraynan VL and Wolpert-Defilippes MK (1986) Synthesis, physical properties and antitumour activity of tetraplatin and related tetrachloroplatinum(IV) stereoisomers of 1,2-diaminocyclohexane. *Cancer Treat Rep* **70**: 997–1002

Appleton TG, Connor JW and Hall JR (1988) S,O- versus S,N- chelation in the reactions of the cis-diamminediaquaplatinum(II) cation with methionine and s-methylcysteine. *Inorg Chem* **27**: 130–137

Borch RF (1987) The platinum anti-tumour drugs. In *Metabolism and Action of Anti-Cancer Drugs*, Powis G and Prough RA (eds), pp 163–193. Taylor and Francis: London

Burchenal JH, Kalaher K, O'Toole T and Chisholm J (1977) Lack of cross-resistance between certain platinum coordination compounds in mouse leukaemia. *Cancer Res* **37**: 3455–3457

Carfagna PF, Poma A, Wyrick SD, Holbrook DJ and Chaney SG (1991) Comparisons of tetrachloro(d,l-trans)1,2-diaminocyclohexane-platinum(IV) biotransformations in the plasma of Fisher 344 rats at therapeutic and toxic doses. *Cancer Chemother Pharmacol* **27**: 335–341

Cavaletti G, Tredici G, Marmiroli P, Petruccioli MG, Barajon I and Fabbrica D (1992) Morphometric study of the sensory neuron and peripheral nerve changes induced by chronic cisplatin (DDP) administration in rats. *Acta Neuropathol* **84**: 364–371

Chaney SG, Wyrick S and Till GK (1990) In vitro biotransformations of tetrachloro(d,l-trans)-1,2-diaminocyclohexaneplatinum(IV) (tetraplatin) in rat plasma. *Cancer Res* **50**: 4539–4545

Clarkson TW (1993) Molecular and ionic mimicry of toxic metals. *Ann Rev Pharmacol Toxicol* **32**: 545–571

De Koning P, Neijt JP, Jennekens FGI and Gispen WH (1987) Evaluation of cis-diamminedichloroplatinum(II) (cisplatin) neurotoxicity in rats. *Toxicol Appl Pharmacol* **89**: 81–87

Dhara SC (1970) A rapid method for the synthesis of cis-[Pt(NH3)2Cl2]. *Indian J Chem* **8**: 193–194

Drayer DE (1986) Pharmacokinetic and pharmacodynamic differences between drug enantiomers in humans: an overview. *Clin Pharm Ther* **40**: 125–133

Eastman A (1987) Glutathione-mediated activation of anticancer platinum(IV) complexes. *Biochem Pharmacol* **36**: 4177–4178

Extra JM, Espie M, Calvo F, Ferme C, Mignot L and Marty M (1990) Phase I study of oxaliplatin in patients with advanced cancer. *Cancer Chemother Pharmacol* **25**: 299–303

Gibbons GR, Wyrick S and Chaney SG (1989) Rapid reduction of tetrachloro(d,l-trans)1,2-diaminocyclohexaneplatinum(IV) (tetraplatin) in RPMI 1640 tissue culture medium. *Cancer Res* **49**: 1402–1407

Gregg RW, Molepa JM, Monpetit VJA, Mikael NZ, Redmond D, Gadia M and Stewart DJ (1992) Cisplatin neurotoxicity: the relationship between dosage, time and platinum concentration in neurological tissues, and morphologic evidence of toxicity. *J Clin Oncol* **10**: 795–803

Hills CA, Kelland LR, Abel G, Siracky J, Wilson AP and Harrap KR (1989) Biological properties of ten human ovarian carcinoma cell lines: calibration in vitro against four platinum complexes. *Br J Cancer* **59**: 527–534

Hoeschele JD, Farrell N, Turner WR and Rithner CD (1988) Synthesis and characterization of diastereomeric (substituted iminodacetato) (1,2-diaminocyclohexane)platinum(II) complexes. *Inorg Chem* **27**: 4106–4113

Kelland LR, Mistry P, Abel G, Loh SY, O'Neill CF, Murrer BA and Harrap KR (1992) Mechanism-related circumvention of acquired cis-diamminedichloroplatinum(II) resistance using two pairs of human ovarian carcinoma cell lines by ammine/amine platinum(IV) dicarboxylates. *Cancer Res* **52**: 3857–3864

Kidani Y, Inagaki K, Iigo M, Hoshi A and Kuretani K (1978) Antitumour activity of 1,2-diaminocyclohexane-platinum complexes against sarcoma-180 ascites form. *J Med Chem* **21**: 1315–1318

Kido Y, Khokhar AR and Siddik ZH (1993) Differential cytotoxicity, uptake and DNA binding of tetraplatin and analogue isomers in sensitive and resistant cancer cell lines. *Anti-Cancer Drugs* **4**: 251–258

Levi F, Misset J, Brienza S, Adam R, Metzger G, Itzakhi M, Caussanel J, Kunstlinger F, Lecouturier S, Descorps-Declere A, Jasmin C, Bismuth H and Reinberg A (1992) A chronopharmacologic phase II clinical trial with 5-fluorouracil, folinic acid and oxaliplatin using an ambulatory multichannel programmable pump: high effectiveness against metastatic colorectal cancer. *Cancer* **69**: 893–900

McKeage MJ, Boxall F, Jones M and Harrap KR (1994) Lack of neurotoxicity of oral bis-acetato-ammine-dichloro-cyclohexylamine-platinum(IV) (JM216) in comparison to cisplatin and tetraplatin in the rat. *Cancer Res* **54**: 629–631

Mauldin SK, Gibbons G, Wyrick SD and Chaney SG (1988) Intracellular biotransformation of platinum compounds with 1,2-diaminocyclohexane carrier ligands in the L1210 cell line. *Cancer Res* **48**: 5136–5144

Pendyala L and Creaven PJ (1993) In vitro cytotoxicity, protein binding, red blood cell partitioning and biotransformation of oxaliplatin. *Cancer Res* **53**: 5970–5976

Petros WP, Chaney SG, Smith DC, Fangmeier J, Sakata M, Brown TD and Trump DL (1994) Pharmacokinetic and biotransformation studies of ormaplatin in conjunction with a phase I clinical trial. *Cancer Chemother Pharmacol* **33**: 347–354

Rowland M and Tozer TN (1989) *Clinical Pharmacokinetics Concepts and Applications*. Lea and Febig: Philadelphia

Schilder RJ, La Creta FP, Perez RP, Johnson SW, Brennan JM, Rogatko A, Nash S, McAleer C, Hamilton TC, Rody D, Young RC, Ozol RF and O'Dwyer PJ (1994) Phase 1 and pharmacokinetic study of ormaplatin (tetraplatin, NSC363812) administered on a day 1 and 8 schedule. *Cancer Res* **54**: 709–717

Shimamura A and Fisher DE (1996) p53 in life and death. *Clin Cancer Res* **2**: 435–440

Siddik ZH, Jones M, Boxall FE and Harrap KR (1988) Comparative distribution and excretion of carboplatin and cisplatin in mice. *Cancer Chemother Pharmocol* **21**: 19–24

Siddik ZH, Al-Baker S, Burditt TL and Khokhar AR (1993) Differential antitumour activity and toxicity of isomeric 1,2-diaminocyclohexane platinum(II) complexes. *J Cancer Res Clin Oncol* **120**: 12–16

© Cancer Research Campaign 1997

Sorenson CM, Barry MA and Eastman A (1990) Analysis of events associated with cell cycle arrest at G2 phase and cell death induced by cisplatin. *J Natl Cancer Inst* **82**: 749–755

Terheggen PMAB, Gerritsen Van Der Hoop R, Floot BGJ and Gispen WH (1989) Cellular distribution of cis-diamminedichloroplatinum(II)-DNA binding in rat dorsal root spinal ganglia: effect of neuroprotecting peptide ORG.766. *Toxicol Appl Pharmacol* **99**: 334–343

Thompson SW, Davies LE, Kornfeld M, Hilgers RD and Standefer JC (1984) Cisplatin neurotoxicity: clinical, electrophysiologic morphologic and toxicologic studies. *Cancer* **54**: 1269–1275

Vollano JF, Al-Baker S, Dabrowiak JC and Schurig JE (1987) Comparative antitumor studies on platinum(II) and platinum(IV) complexes containing 1,2-diaminocyclohexane. *J Med Chem* **30**: 716–719

Walton MI, Wu E, Koshy P, Sharp SY and Kelland LR (1996) p53 status and CDDP sensitivity in a panel of ovarian cancer cell lines. *Proc Am Assoc Cancer Res* **37**: 402

Wilkoff LJ, Dulmadge EA, Trader MW, Harrison SD and Griswold DP (1987) Evaluation of trans-tetrachloro-1,2-diaminocyclohexane platinum(IV) in murine leukaemia L1210 resistant and sensitive to cis-diamminedochloroplatinum(II). *Cancer Chemother Pharmacol* **20**: 96–100

© Cancer Research Campaign 1997